# Effect of transcranial direct current stimulation with concurrent cognitive performance targeting posterior parietal cortex vs prefrontal cortex on working memory in schizophrenia: a randomized clinical trial

**DOI:** 10.1038/s41398-024-02994-w

**Published:** 2024-07-08

**Authors:** Wenpeng Hou, Fuchun Zhou, Qi Wang, Hang Li, Xiangqin Qin, Yushen Ding, Fang Dong, Qijing Bo, Anning Li, Liang Zhang, Zhenzhu Chen, Zhimin Wang, Xianbin Li, Jimmy Lee, Chuanyue Wang

**Affiliations:** 1 Beijing Key Laboratory of Mental Disorders, National Clinical Research Center for Mental Disorders & National Center for Mental Disorders, Beijing Anding Hospital, Capital Medical University, Beijing, China; 2 Advanced Innovation Center for Human Brain Protection, Capital Medical University, Beijing, China; 3 Fengtai Mental Health Center, Beijing, China; 4 Institute of Mental Health, Singapore, Singapore; 5 Lee Kong Chian School of Medicine, Nanyang Technological University, Singapore, Singapore

**Keywords:** Schizophrenia, Molecular neuroscience

## Abstract

Working memory deficits are linked to irregularities in the dorsolateral prefrontal cortex (DLPFC) and the posterior parietal cortex (PPC) in schizophrenia, effective intervention strategies are lacking. We evaluated the differential efficacy and underlying neuromechanisms of targeting transcranial direct current stimulation (tDCS) at the DLPFC and the PPC with concurrent cognitive performance for working memory in schizophrenia. In a randomized and double-blind clinical trial, sixty clinically stable schizophrenic patients with below-average working memory were randomly assigned to active DLPFC, active PPC, and sham tDCS groups. Two sessions of tDCS during N-back task were delivered daily for five days. The primary outcome was changes in spatial span test scores from baseline to week 1. The secondary outcomes included changes in scores of color delay-estimation task, other cognitive tasks, and mismatch negativity (biomarker of N-methyl-d-aspartate receptor functioning). Compared with the active DLPFC group, the active PPC group demonstrated significantly greater improvement in spatial span test scores (*p* = 0.008, *d* = 0.94) and an augmentation in color delay-estimation task capacity at week 1; the latter sustained to week 2. Compared with the sham tDCS group, the active PPC group did not show a significant improvement in spatial span test scores at week 1 and 2; however, significant enhancement was observed in their color delay-estimation task capacity at week 2. Additionally, mismatch negativity amplitude was enhanced, and changes in theta band measures were positively correlated with working memory improvement in the active PPC group, while no such correlations were observed in the active DLPFC group or the sham tDCS group. Our results suggest that tDCS targeting the PPC relative to the DLPFC during concurrent cognitive performance may improve working memory in schizophrenia, meriting further investigation. The improvement in working memory appears to be linked to enhanced N-methyl-d-aspartate receptor functioning.

## Introduction

Working memory is the brain’s ability to hold and manipulate transient information necessary for complex cognitive tasks [1]. Deficits in working memory constitute a primary aspect of cognitive impairment in patients with schizophrenia, often severely affecting their daily functioning [2]. However, effective treatments for the deficits in working memory in schizophrenia are notably lacking [3–5]. Transcranial direct current stimulation (tDCS) is capable of modulating subthreshold membrane potentials, thereby altering the likelihood of action potentials, affecting the activation, neural oscillations, and functional interactions of brain regions [6, 7]. Consequently, tDCS may represent a viable therapeutic strategy for ameliorating working memory deficits in schizophrenia.

Both the dorsolateral prefrontal cortex (DLPFC) and the posterior parietal cortex (PPC) are associated with the execution of working memory [8–12]. Although the majority of tDCS research on schizophrenia has targeted the DLPFC, yielding mixed results according to extant meta-analyses [13–15], no studies have yet targeted the PPC in patients with schizophrenia, nor have any studies directly compared the effects of tDCS on the DLPFC and the PPC in this patient population. However, a study highlighted the superior effect of targeting the PPC over the DLPFC with single-session tDCS in bolstering working memory performance among healthy young individuals [16]. In contrast, other studies have reported conflicting results [17–19]. These inconsistencies might be due to differences in assessment tools and the subtle effects of single-session stimulation.

The effect of tDCS may be task specific due to the requirement for plasticity induction in the targeted neural pathway [20, 21]. Thus, a transcranial electrical stimulation workshop [20] recommended tDCS to be performed during task execution to enhance modulation effects, yet preliminary studies reported inconclusive results [22–27]. The inconclusive results might be related to the fact that existing studies mainly targeted the DLPFC, which had shown mixed cognitive improvement effects. Given that the PPC is also related to working memory performance, it is worthwhile to investigate the potential utility of the PPC as an alternative stimulation target when combining tDCS with concurrent working memory task to enhance working memory in schizophrenia.

The elicitation of tDCS effects necessitates the induction of endogenous N-methyl-d-aspartate receptor (NMDAR) [28]. The NMDAR is also pivotal for cortical activation and synaptic plasticity [29, 30]. Mismatch negativity (MMN) acts as a neurophysiological index for automatic sensory deviation detection and is sensitive to NMDAR agonists [31]. MMN is an effective biomarker of NMDAR deficits in schizophrenia [32, 33], and an indicator of theta band features [34]. Among patients with schizophrenia, there are notable reductions in MMN amplitude, theta band power, and theta band intertrial coherence [35, 36]. Research suggests that MMN can potentially be modulated by targeting specific brain regions with tDCS [37–41]. Additionally, D-serine acts as an endogenous co-agonist of NMDAR [29] and is synthesized in both brain and peripheral tissues [42, 43]. Because D-serine can traverse the blood-brain barrier, its serum levels correlate with its concentration in the brain in animal studies [44]. Hence, both MMN and serum D-serine level can serve as surrogate biomarkers for NMDAR functioning.

Overall, no study has compared the efficacy of tDCS targeting the DLPFC or the PPC during concurrent cognitive task for addressing working memory deficits in schizophrenia. We hypothesized that tDCS targeting the PPC would be more effective than targeting the DLPFC during concurrent working memory task for working memory in schizophrenia. To test this hypothesis, we conducted a randomized controlled clinical trial and utilized biomarkers of the NMDAR (i.e., MMN and D-serine) to attempt to disentangle the intrinsic mechanisms for the therapeutic efficacy.

## Methods and materials

### Study overview

This single-center, three-arm, double-blind (participant and assessor blind) clinical trial was conducted at the Beijing Anding Hospital affiliated with Capital Medical University in China, from September 2021 to May 2023. This trial was registered in the Chinese Clinical Trial Registry (ChiCTR2000038961), and the protocol was approved by the Ethics Committee of the Beijing Anding Hospital. Participants signed informed consent forms after being fully informed of the trial.

### Participants

Participants were outpatients recruited via advertisements, community and outpatient physician referrals. The inclusion criteria included a diagnosis of schizophrenia based on DSM-5 criteria using the Mini International Neuropsychiatric Interview (MINI 7.0.2), aged 18–50 years, right-handed, at least 8 years of education, intelligence quotient (IQ) > 70, and a spatial span test T-score of 30–50. Participants were clinically stable [45], with Positive and Negative Syndrome Scale (PANSS) scores not exceeding 5 on any of the following items: P1 delusion, P3 hallucination, P5 exaggeration, P6 suspicion/victimization, and G9 abnormal thinking content; scored no more than 4 on P2 concept disorder; stayed on current antipsychotic and adjunctive medications for a minimum of 6 weeks, with a fixed antipsychotic dose for at least 2 weeks. Throughout the study, the type and dose of antipsychotic medications remained unchanged. The rationale behind selecting participants with a spatial span test score of 30–50 was based on a computerized cognitive remediation therapy study [46, 47] involving 311 schizophrenic patients, which demonstrated that participants with baseline cognitive impairment of 1–2 standard deviations reaped the most cognitive benefits. The exclusion criteria were detailed in the Supplementary Methods. The study also included healthy controls who were matched with the patient group in terms of sex, age, and educational level. Eligibility criteria for the controls included an age range of 18–50 years, right-handedness, and a minimum education duration of 8 years; an IQ above 70 was required. Additionally, the controls must have had no diagnosed psychiatric disorders, neurological conditions, or other severe physical illnesses, and no family history of psychiatric disorders.

### Design

A balanced 1:1:1 allocation was accomplished through block randomization, with allocation concealment implemented using the envelope method. This step resulted in participants being randomly allocated to one of the three groups: an active DLPFC group, an active PPC group, and a sham tDCS group. In the sham tDCS condition, electrode placement was designed to mirror that of active PPC group in half of the subjects and identical to that of active DLPFC group in the other half, consistent with the design in research on healthy individuals [16]. Following the protocol by Valiengo et al. [48], 2 tDCS sessions were conducted daily, with a gap of at least 3 h, over a 5-day period (10 sessions). In all three groups, participants underwent a 20-min visuospatial N-back task [49] during each session. Assessments were conducted by one psychiatrist and two psychologists blind to the patients’ group assignment at baseline, week 1 (the 2nd to 3rd day following the last intervention) and week 2 (the 8th to 9th day following the last intervention). Additionally, healthy controls were included in this study primarily to facilitate the description of impairments and improvements in spatial span test scores across the three patient groups.

### Intervention

The high-definition tDCS was administered using a 4 × 1 wire adaptor (Equalizer Box, NeuroConn, Germany) connected to a constant current stimulator (DC-Stimulator Plus, NeuroConn, Germany). Because high-definition tDCS offers a higher spatial focality [20] and more robust and durable modulation of cortical plasticity [50] than conventional tDCS, it is more suitable for investigations on the differential effects of stimulating different brain regions. Considering the abnormalities in the left frontal-parietal cortex during verbal and visuospatial working memory tasks in schizophrenia [9, 11, 51], and the predominant selection of the left hemisphere as the anodal stimulation target in tDCS studies on schizophrenia [13], this study chose the left hemisphere for stimulation targets. The cap based on the EEG 10-5 system was used for localization. For the left DLPFC target, F3 served as the central electrode, with F7, Fz, Fp1, FC3 functioning as surrounding electrodes. For the left PPC target, P3 was chosen as the central electrode, while P7, Pz, O1, CP3 were selected as surrounding electrodes. FC3 and CP3 were chosen to minimize possible effects of active DLPFC on the PPC and active PPC on the DLPFC, respectively. Electrical field simulations were performed using ROAST 3.0 [52] (Supplementary Fig. 2). For the two active stimulation groups, the current intensity was 2 mA for 20 min, with a 40-s ramp-up and ramp-down period. Sham tDCS mirrored this setup, but the real stimulation duration was limited to 40 s.

### Outcomes and assessments

The spatial span test is the sole task for working memory in the Chinese version of the MATRICS Consensus Cognitive Battery (MCCB) [53] which is widely recognized as the gold standard for cognitive assessments in clinical trials for schizophrenia [45]. Thus, the primary outcome was changes in spatial span test scores at week 1 from baseline. The secondary outcomes included changes in spatial span test scores at week 2, changes in scores of the color delay-estimation task, digit sequencing task [54], Stroop task, MCCB scales, PANSS [53] scales, Calgary Depression Scale for Schizophrenia (CDSS), MMN and tDCS adverse effects. The standardized T scores were used for the MCCB. d-serine level changes were considered an exploratory outcome.

To evaluate the capacity and precision of visual working memory simultaneously, we implemented the color delay-estimation task as described by Zhao et al. [55]. To assess attention control, we used the Stroop with adaptive response deadline adapted by Draheim et al. [56, 57]. Adverse effects of tDCS were recorded at the end of each treatment session and in the follow-up assessments at week 1 and week 2 using the tDCS Adverse Effects Questionnaire [58]. To evaluate the integrity of the blinding, participants were requested to speculate on whether they had received real or sham stimulation upon completing the week 2 assessment. Details of these tasks are provided in Supplementary Methods.

### Biomarkers

We employed an Oddball paradigm, comprised of 90% standard stimuli and 10% duration-deviant stimuli, to elicit the MMN. For each participant, the MMN amplitude was the peak negative amplitude between 140 and 240 ms from the waveform. The waveform was derived from subtracting the average waveform of the standard stimuli from that of the deviant stimuli. MMN theta power and theta intertrial coherence were defined as the peak mean power and intertrial coherence within the theta band (4–7 Hz) during the 140–240 ms interval following the deviant stimulus. Peripheral serum concentrations of D-serine were obtained using the enzyme-linked immunosorbent assay technique. Details are provided in Supplementary Methods.

### Statistical analysis

In a study examining the impact of single-session tDCS on working memory in healthy young individuals, the PPC stimulation group showed an effect size of 0.7 compared to the DLPFC stimulation group [16]. Given that our study focused on schizophrenia patients with working memory deficits and employed multi-session tDCS with concurrent cognitive performance, we estimated a larger differential effect size between the DLPFC and PPC stimulation groups, setting it at 1.0. Test power was set at 80%, with a two-tailed α level of 5%. This yielded 17 patients for each of the two groups. Accounting for a potential dropout rate of 15%, we required 20 participants in each group, with additional 20 patients included in the sham stimulation group as a control.

In the intention-to-treat sample, statistical analysis was conducted using SPSS 20.0 (SPSS Inc., Chicago, IL, USA). Histograms and Shapiro-Wilk tests were used to assess the normality of data distribution. For the data that were not normally distributed, logarithmic transformations or non-parametric tests were applied to address the issue. First, when baseline characteristics, chlorpromazine equivalents of antipsychotic medications [59] and adverse effects were compared across the three groups, chi-square tests or Fisher’s exact tests were employed for categorical variables, whereas one-way analysis of variance and Kruskal-Wallis tests were utilized for continuous variables. Second, Chi-square tests or Fisher’s exact tests were applied to compare the number of participants within each group who guessed they were assigned to the real stimulation group, as an assessment of blinding integrity.

Third, the primary efficacy analysis was conducted with linear mixed models to compare changes in spatial span test scores over time (from week 1 to baseline) among the three groups. To delve deeper into the changes of the primary outcome, we have also compared the changes at week 2 from baseline and examined the changes across all three time points among the three groups. The secondary efficacy analyses encompassed comparisons of changes in cognitive performance, clinical symptoms, and MMN across the three groups over specified timepoints (baseline, week 1, and week 2). Additionally, an exploratory analysis was conducted on d-serine levels. Healthy controls underwent the MCCB assessment to characterize baseline impairments in various cognitive domains within the patient groups, as well as to quantify the degree of improvement in spatial span test scores post-intervention. The analysis was performed using independent sample t-tests.

Last, given that the effects of interventions on biomarkers might be immediate, Spearman correlation analysis was used to examine the relationship between changes in MMN and D-serine levels at week 1 from baseline and changes in spatial span test scores at week 1 and 2 from baseline within each group. False discovery rate (FDR) corrections were applied across the groups. The goal was to elucidate possible underlying mechanisms of therapeutic efficacy.

A *p*-value < 0.05 (two-sided) was deemed statistically significant. Informed by Wobrock et al. [60], effect sizes were calculated based on changes in scores from the baseline for each group using an online tool [61]. Cohen’s *d* values indicate small (0.2–0.5), medium (0.5–0.8), and large (≥0.8) effect sizes [62].

## Results

### Participants

Of the 60 participants, 54 (90%) completed the assessment at week 1, and 52 (87%) completed the week 2 assessment (Supplementary Fig. 1). No significant differences were observed across the groups in baseline characteristics except for speed of processing domain in MCCB (*F*_2, 57_ = 3.65, *p* = 0.03) (Table 1). Thirty-five healthy controls were included in the study. Compared with these controls, the patient group exhibited significantly lower IQ and MCCB scores across all domains at baseline (*p* < 0.05) (Supplementary Table 1).

**Table 1:** Baseline characteristics of the participants.

Characteristic	tDCS Group, Mean (SD)					
	Active DLPFC (n = 20)	Active PPC (n = 20)	Sham tDCS (n = 20)	Statistic^d^	*df*	*p*
Women, No. (%)	9 (45)	14 (70)	11 (55)	*χ²* = 2.58	2	0.28
Han nationality, No. (%)	19 (95)	18 (90)	18 (90)	*χ²* = 0.62	NA	>0.99
Unemployed, No. (%)	12 (60)	12 (60)	12 (60)	*χ²* < 0.001	2	>0.99
Not married, No. (%)	13 (65)	12 (60)	13 (65)	*χ²* = 0.14	2	>0.99
Current smoking, No. (%)	3 (15)	3 (15)	1 (5)	*χ²* = 1.37	NA	0.68
Clozapine use, No. (%)	2 (10)	4 (20)	4 (20)	*χ²* = 1.04	NA	0.75
ECT history, No. (%)	4 (20)	8 (40)	8 (40)	*χ²* = 2.40	2	0.34
Age, y	32.10 (7.44)	34.10 (7.41)	33.45 (7.71)	*F* = 0.37	2, 57	0.69
Education, y	15.15 (3.62)	14.40 (3.24)	14.38 (3.88)	*F* = 0.30	2, 57	0.74
IQ	104.81 (11.30)	104.05 (9.69)	103.41 (10.96)	*F* = 0.09	2, 57	0.92
Duration of illness, y	8.41 (7.21)	13.04 (8.51)	11.42 (7.80)	*H* = 3.65	2	0.16
No. of hospitalizations	1.55 (2.19)	2.35 (2.16)	1.95 (1.96)	*H* = 3.28	2	0.19
Duration of current antipsychotic medication type, m	40.23 (55.48)	36.90 (41.71)	46.35 (68.41)	*H* = 0.11	2	0.95
Duration of current antipsychotic medication dose, m	20.20 (38.65)	14.44 (15.87)	14.05 (19.79)	*H* = 0.32	2	0.85
Chlorpromazine equivalents, mg/day	421.53 (344.91)	437.20 (266.77)	429.25 (253.34)	*H* = 0.58	2	0.75
PANSS Positive	9.10 (4.22)	9.35 (2.91)	8.25 (2.59)	*F* = 0.61	2, 57	0.55
PANSS Negative	13.00 (4.43)	10.90 (3.32)	13.35 (5.30)	*F* = 1.79	2,0	0.18
PANSS General	22.00 (5.09)	21.35 (4.44)	19.60 (3.44)	*F* = 1.61	2, 57	0.21
PANSS Total	44.10 (10.37)	41.60 (7.62)	41.20 (8.17)	*F* = 0.64	2, 57	0.53
CDSS score	2.35 (2.81)	4.25 (4.23)	2.70 (3.47)	*H* = 2.70	2	0.26
Spatial span test	41.75 (5.13)	41.85 (5.21)	41.10 (5.48)	*F* = 0.12	2, 57	0.89
Delay-estimation task capacity^a^	1.98 (0.75)	1.79 (0.64)	2.18 (0.82)	*F* = 1.40	2, 55	0.25
Delay-estimation task precision^a^	0.07 (0.04)	0.09 (0.08)	0.06 (0.04)	*H* = 4.09	2	0.13
Digit sequencing task	21.45 (4.01)	22.10 (4.25)	22.75 (3.60)	*F* = 0.54	2, 57	0.59
StroopDL response time	907.18 (368.43)	751.01 (238.12)	869.53 (405.19)	*F* = 1.12	2, 57	0.33
MCCB speed of processing	33.25 (12.34)	39.85 (9.17)	41.70 (9.38)	*F* = 3.65	2, 57	0.03
MCCB attention	45.90 (11.96)	46.85 (9.96)	48.00 (9.59)	*F* = 0.20	2, 57	0.82
MCCB verbal learning	36.40 (10.90)	38.90 (10.97)	38.75 (9.66)	*F* = 0.35	2, 57	0.70
MCCB visual learning	41.60 (9.66)	43.25 (10.76)	40.90 (9.78)	*F* = 0.29	2, 57	0.75
MCCB reasoning and problem solving	41.60 (11.11)	47.55 (11.46)	43.45 (10.03)	*F* = 1.56	2, 57	0.22
MCCB neurocognitive composite	35.15 (11.41)	39.55 (8.47)	38.55 (6.82)	*F* = 1.28	2, 57	0.28
MMN amplitude, μV^b^	−3.01 (1.14)	-2.46 (1.25)	−3.42 (2.24)	*H* = 2.96	2	0.23
MMN theta power^b^	3.58 (1.36)	3.25 (1.07)	3.54 (1.48)	*H* = 0.28	2	0.87
MMN theta intertrial coherence^b^	0.18 (0.13)	0.20 (0.11)	0.19 (0.14)	*H* = 0.21	2	0.90
D-serine, ng/ml^c^	70.00 (39.55)	81.02 (44.22)	64.43(16.93)	*H* = 1.46	2	0.48

*ECT* electroconvulsive therapy, *IQ*, intelligence quotient, PANSS Positive and Negative Syndrome Scale, *CDSS* Calgary Depression Scale for Schizophrenia, *StroopDL* Stroop with adaptive response deadline, *MCCB* MATRICS Consensus Cognitive Battery, *MMN* mismatch negativity, *DLPFC* dorsolateral prefrontal cortex, *PPC* posterior parietal cortex, *tDCS* transcranial direct current stimulation.

^a^For color delay-estimation task, the sample size was 19 (active DLPFC), 20 (active PPC) and 19 (sham tDCS).

^b^For MMN, the sample size was 20 (active DLPFC), 20 (active PPC) and 19 (sham tDCS).

^c^For d-serine, the sample size was 19 (active DLPFC), 17 (active PPC) and 19 (sham tDCS).

^d^*χ²* means chi-square tests or Fisher’s exact tests, *F* means one-way analysis of variance, *H* means Kruskal-Wallis tests.

### Primary outcome: spatial span test scores

**Fig. 1:**
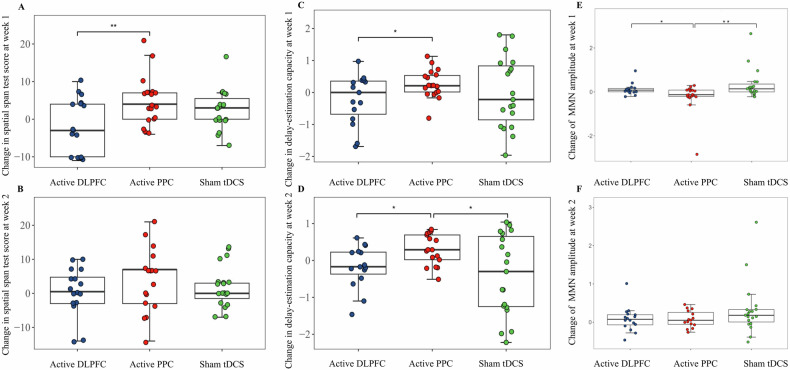
Changes in spatial span test score, delay-estimation task capacity and MMN amplitude. Changes in spatial span test score from baseline to week 1 (**A**) and week 2 (**B**) across the three groups. Changes in delay-estimation task capacity from baseline to week 1 (**C**) and week 2 (**D**). Changes in MMN amplitude from baseline to week 1 (**E**) and week 2 (**F**). MMN mismatch negativity, DLPFC dorsolateral prefrontal cortex; PPC, posterior parietal cortex; tDCS, transcranial direct current stimulation. MMN amplitude was In-transformed. *p < 0.05, ** *p* < 0.01.

A significant time-by-group interaction was found in spatial span test scores from week 1 to baseline across the three groups (*F*_2, 51_ = 4.66, *p* = 0.01) (Table 2). Specifically, the active PPC group demonstrated significant improvement in the spatial span test scores than the active DLPFC group at week 1 (*F*_1, 34_ = 7.95, *p* = 0.008, *d* = 0.94) (Table 2) (Fig. 1A) (Supplementary Figure 3). However, no significant time-by-group interaction was found in spatial span test scores over three time points (baseline, week 1, week 2) across the three groups (Table 3).

**Table 2:** Effects on cognition.

Outcome^a^	tDCS Group, Mean (SD)			*p*	Active DLPFC vs Active PPC		Active DLPFC vs Sham tDCS		Active PPC vs Sham tDCS	
	Active DLPFC (n = 20)	Active PPC (n = 20)	Sham tDCS (n = 20)		*p*	Cohen’s *d* (95% CI)	*p*	Cohen’s *d* (95% CI)	*p*	Cohen’s *d* (95% CI)
Primary outcome										
Change in spatial span test score at week 1	−1.56 (7.28)	4.89 (6.44)	2.63 (5.28)	0.01	0.008	0.94 (0.24 to 1.65)	0.06	0.67 (−0.02 to 1.35)	0.24	−0.38 (−1.03 to 0.26)
*Secondary Outcomes*										
Change in score at week 1										
Delay-estimation task capacity ^*b*^	−0.22 (0.78)	0.27 (0.44)	−0.01 (1.10)	0.30	0.04	0.80 (0.10 to 1.50)	0.67	0.22 (−0.46 to 0.90)	0.32	−0.33 (−0.98 to 0.31)
Delay-estimation task precision (In-transformed)^*b*^	0.001 (0.007)	−0.002 (0.008)	0 (0.005)	0.38	0.21	−0.40 (−1.08 to 0.29)	0.41	−0.17 (−0.85 to 0.51)	0.50	0.30 (−0.34 to 0.94)
Digit sequencing task	1.44 (3.05)	1.05 (2.04)	1.42 (3.02)	0.84	0.59	−0.15 (−0.82 to 0.51)	0.95	−0.01 (−0.67 to 0.66)	0.61	0.14 (−0.49 to 0.78)
StroopDL response time ^*c*^	−70.63 (209.49)	−98.03 (243.30)	−82.68 (247.88)	0.96	0.99	−0.12 (−0.80 to 0.56)	0.75	−0.05 (−0.73 to 0.63)	0.87	0.06 (−0.57 to 0.70)
MCCB speed of processing	6.44 (5.91)	6.11 (3.97)	4.11 (6.14)	0.39	0.85	0.07 (−0.73 to 0.60)	0.28	−0.39 (−1.06 to 0.29)	0.25	−0.39 (−1.03 to 0.26)
MCCB attention	1.75 (7.69)	2.32 (6.64)	0.79 (6.28)	0.78	0.96	0.08 (−0.59 to 0.75)	0.72	−0.14 (−0.80 to 0.53)	0.47	−0.24 (−0.88 to 0.40)
MCCB verbal learning	1.44 (13.19)	0.84 (9.82)	1.37 (11.77)	0.95	0.75	−0.05 (−0.72 to 0.61)	0.84	−0.01 (−0.67 to 0.66)	0.89	0.05 (−0.59 to 0.69)
MCCB visual learning	0.56 (9.13)	2.37 (8.63)	1.84 (9.66)	0.91	0.63	0.20 (−0.46 to 0.87)	0.75	0.14 (−0.53 to 0.80)	0.96	−0.06 (−0.69 to 0.58)
MCCB reasoning and problem solving	7.19 (9.87)	3.63 (6.98)	4.32 (7.43)	0.34	0.19	−0.42 (−1.10 to 0.25)	0.27	−0.33 (−1.00 to 0.34)	0.82	0.10 (−0.54 to 0.73)
MCCB neurocognitive composite	4.00 (6.47)	5.05 (5.03)	3.63 (5.96)	0.76	0.64	0.18 (−0.48 to 0.85)	0.79	−0.06 (−0.73 to 0.61)	0.43	−0.26 (−0.90 to 0.38)
Change in score at week 2										
Spatial span test	0.50 (7.13)	3.88 (9.23)	2.05 (6.16)	0.46	0.26	0.41 (−0.28 to 1.10)	0.49	0.23 (−0.43 to 0.90)	0.52	−0.24 (−0.89 to 0.42)
Delay-estimation task capacity ^*d*^	−0.17 (0.57)	0.29 (0.42)	-0.40 (1.09)	0.04	0.02	0.93 (0.20 to 1.66)	0.33	−0.26 (−0.94 to 0.42)	0.02	−0.82 (−1.50 to -0.14)
Delay-estimation task precision (In-transformed) ^*d*^	0.006 (0.02)	−0.002 (0.009)	0.001 (0.005)	0.29	0.21	−0.53 (−1.23 to 0.18)	0.42	−0.36 (−1.05 to 0.32)	0.16	0.42 (−0.24 to 1.08)
Digit sequencing task	2.06 (3.42)	1.76 (2.08)	1.63 (3.11)	0.80	0.52	−0.11 (−0.79 to 0.58)	0.66	−0.13 (−0.80 to 0.53)	0.85	−0.05 (−0.70 to 0.61)
StroopDL response time	−93.88 (229.79)	−82.46 (232.51)	−156.14(353.28)	0.71	0.67	0.05 (−0.63 to 0.73)	0.76	−0.21 (−0.87 to 0.46)	0.42	−0.24 (−0.90 to 0.41)
MCCB speed of processing	9.94 (8.53)	7.76 (5.85)	7.26 (6.36)	0.48	0.33	−0.30 (−0.99 to 0.39)	0.31	−0.36 (−1.03 to 0.31)	0.89	−0.08 (−0.74 to 0.57)
MCCB attention	3.44 (8.94)	2.71 (6.56)	4.42 (6.12)	0.74	0.66	−0.09 (−0.78 to 0.59)	0.85	0.13 (−0.54 to 0.80)	0.40	0.27 (−0.39 to 0.93)
MCCB verbal learning	7.75 (9.90)	10.12 (7.77)	5.84 (8.36)	0.37	0.60	0.27 (-0.42 to 0.95)	0.44	−0.21 (−0.88 to 0.46)	0.14	−0.53 (−1.20 to 0.14)
MCCB visual learning	13.19 (8.73)	10.00 (8.74)	6.47 (12.14)	0.13	0.21	−0.37 (−1.05 to 0.32)	0.06	−0.63 (−1.31 to 0.05)	0.42	−0.33 (−0.99 to 0.33)
MCCB reasoning and problem solving	7.13 (8.92)	5.24 (7.08)	7.32 (8.08)	0.63	0.39	−0.24 (−0.92 to 0.45)	0.96	0.02 (−0.64 to 0.69)	0.39	0.27 −0.39 to 0.93)
MCCB neurocognitive composite	10.56 (7.52)	10.06 (4.28)	8.16 (6.11)	0.44	0.65	−0.08 (−0.77 to 0.60)	0.25	−0.35 (−1.02 to 0.32)	0.30	−0.36 (−1.02 to 0.30)

*tDCS* transcranial direct current stimulation, *StroopDL* Stroop with adaptive response deadline, *MCCB* MATRICS Consensus Cognitive Battery, *DLPFC* dorsolateral prefrontal cortex, *PPC* posterior parietal cortex.

^a^For most tasks at week 1, the sample size was 16 (active DLPFC), 19 (active PPC), and 19 (sham tDCS). For most tasks at week 2, the sample size was 16 (active DLPFC), 17 (active PPC) and 19 (sham tDCS).

^b^For color delay-estimation task at week 1, the sample size was 15 (active DLPFC), 19 (active PPC) and 19 (sham tDCS).

^c^For StroopDL at week 1, the sample size was 15 (active DLPFC), 19 (active PPC) and 19 (sham tDCS).

^d^For color delay-estimation task at week 2, the sample size was 15 (active DLPFC), 17 (active PPC) and 19 (sham tDCS).

**Table 3:** Linear mixed models over three visit points across three groups.

	Time			Group			Time-by-group Interaction		
Outcome	*F*	*df*	*p*	*F*	*df*	*p*	*F*	*df*	*p*
Spatial span test	7.01	1, 52	0.01	0.48	2, 56	.62	1.74	2, 52	0.19
Delay-estimation task capacity	1.15	1, 52	0.29	3.43	2, 54	.04	4.04	2, 52	0.02
Delay-estimation task precision (In-transformed)	0.23	1, 57	0.63	1.55	2, 56	.22	1.53	2, 57	0.23
Digit sequencing task	22.57	1, 55	<0.001	0.56	2, 57	.57	0.20	2, 55	0.82
StroopDL response time	11.33	1, 52	0.001	0.90	2, 56	.41	0.31	2, 52	0.74
MCCB speed of processing	83.85	1, 51	<0.001	3.69	2, 55	.03	0.69	2, 51	0.51
MCCB attention	13.69	1, 51	0.001	0.13	2, 56	.88	0.28	2, 51	0.75
MCCB verbal learning	70.07	1, 51	<0.001	0.70	2, 56	.50	1.83	2, 51	0.17
MCCB visual learning	54.53	1, 52	<0.001	0.52	2, 56	.60	2.06	2, 52	0.14
MCCB reasoning and problem solving	38.46	1, 52	<0.001	1.27	2, 56	.29	0.37	2, 52	0.70
MCCB neurocognitive composite	146.38	1, 50	<0.001	1.50	2, 55	.23	0.99	2, 50	0.38
PANSS Positive	0.25	1, 42	0.62	0.18	2, 53	.83	0.58	2, 42	0.56
PANSS Negative	13.20	1, 49	0.001	3.00	2, 55	.06	3.19	2, 49	0.0498
PANSS General	8.23	1, 49	0.006	2.19	2, 55	.12	2.91	2, 49	0.06
PANSS total	13.80	1, 43	0.001	1.13	2, 54	.33	2.81	2, 42	0.07
MMN amplitude (In-transformed)	8.73	1, 57	0.005	1.10	2, 56	.34	1.20	2, 57	0.31
MMN theta power (In-transformed)	21.13	1, 52	<0.001	0.57	2, 56	.57	0.54	2, 52	0.58
MMN theta intertrial coherence (In-transformed)	1.91	1, 51	0.17	0.07	2, 54	.93	0.34	2, 51	0.71
d-serine (In-transformed)	2.56	1, 44	0.12	0.65	2, 51	.53	0.49	2, 44	0.61

*StroopDL* Stroop with adaptive response deadline, *MCCB* MATRICS Consensus Cognitive Battery, *PANSS* Positive and Negative Syndrome Scale, *MMN* mismatch negativity.

**Fig. 2:**
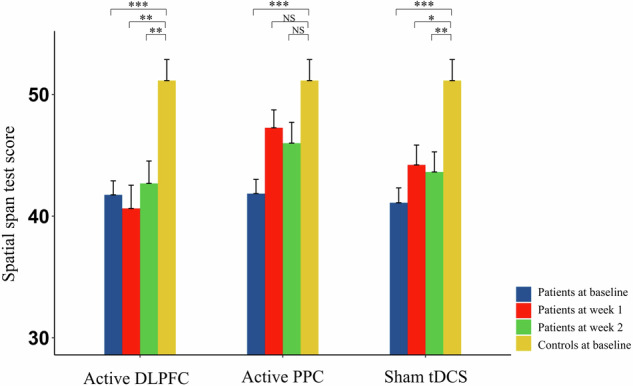
Comparison of spatial span test scores between three patient groups and healthy controls before and after intervention. DLPFC dorsolateral prefrontal cortex, PPC posterior parietal cortex, tDCS transcranial direct current stimulation. *p < 0.05, ** *p* < 0.01, *** *p* < 0.001, NS not significant.

At week 1 and 2, the spatial span test scores for both the active DLPFC group and the sham stimulation group remained significantly lower than those of the healthy controls (*p* < 0.05). However, the scores of the active PPC group approached those of the healthy controls, with no significant differences between the two groups (Fig. 2).

### Secondary outcome: cognition

There was a significant time-by-group interaction in color delay-estimation task capacity over time (baseline, week 1, week 2) across the three groups (*F*_*2, 52*_ = 4.04, *p* = 0.02) (Table 3). Specifically, compared to the active DLPFC group, the active PPC group showed significantly greater augmentation in color delay-estimation task capacity at week 1 (*F*_1, 33_ = 4.68, *p* = 0.04, *d* = 0.80), and this effect persisted at week 2 (*F*_1, 31_ = 5.91, *p* = .02, *d* = 0.93). Color delay-estimation task capacity at week 2 was also higher in the active PPC group vs the sham stimulation group (*F*_1, 37_ = 5.80, *p* = .02, *d* = −0.82). There were no significant differences in the changes in color delay-estimation task capacity between the active DLPFC group and the sham stimulation group (Table 2 and Fig. 1C, D). No time-by-group interactions were found for any other cognitive measures (Tables 2, 3 and Fig. 1B).

### Secondary outcome: clinical symptoms

There was a significant time-by-group interaction in negative symptoms over time (baseline, week 1, week 2) across the three groups (*F*_*2, 49*_ = 3.19, *p* = 0.0498) (Table 3). Specifically, there was significant reduction in negative symptoms at week 2 in the active DLPFC group compared to the active PPC group (*F*_1, 31_ = 4.80, *p* = .04, *d* = 0.80). The sham stimulation group also exhibited greater improvement in negative symptoms at week 2 compared to the active PPC group (*F*_1, 32_ = 7.82, *p* = 0.009, *d* = −0.93). There were no significant differences in the changes in negative symptoms between the active DLPFC group and the sham stimulation group (Supplementary Table 2).

The cognitive and clinical outcomes remained consistent with the above results, after accounting for sex, clozapine use, electroconvulsive therapy history, duration of illness, speed of processing and MCCB neurocognitive composite score as covariates in the linear mixed models.

### Biomarker: MMN

The active PPC group demonstrated improvement (more negative) in MMN amplitude at week 1 compared to both the active DLPFC group (*p* = 0.04, *d* = −0.75) and the sham stimulation group (*p* = 0.006, *d* = 0.93). No significant differences were observed in MMN amplitude changes between the active DLPFC group and the sham stimulation group (Supplementary Table 3, Fig. 1E and Supplementary Fig. 4).

### Exploratory outcome: d-serine

There were no significant time-by-group interactions in changes of d-serine levels over time across the three groups (Table 3) (Supplementary Table 3).

Correlation between Change in Mismatch Negativity, d-serine and Change in Working Memory

In the active DLPFC group, no significant correlations were observed between the changes in MMN indices and D-serine levels at week 1 from baseline and the changes in spatial span test scores at week 1 and 2 from baseline (*p* > 0.05 after FDR correction) (Supplementary Fig. 5A, Supplementary Table 5).

Conversely, in the active PPC group, the change in MMN theta intertrial coherence at week 1 positively linked to the changes in the spatial span test scores at week 2 (*r* = 0.89, *p* < 0.001 after FDR correction). Within this group, there were no significant associations between the changes in other MMN indices or d-serine levels and the changes in the spatial span test scores (Supplementary Fig. 5B, Supplementary Table 5).

In the sham stimulation group, no significant correlations were found between the changes in MMN indices and d-serine levels at week 1 from baseline and the changes in spatial span test scores at week 1 and 2 from baseline (*p* > 0.05 after FDR correction) (Supplementary Fig. 5C, Supplementary Table 5).

In the total sample at baseline, neither d-serine level nor MMN measures were correlated with the spatial span test score or delay-estimation task capacity.

### Adverse effects and integrity of blinding

Adverse effects of tDCS after the 10^th^ treatment session are detailed in Supplementary Table 4. The active DLPFC group reported higher levels of scalp pain, tingling, and burning sensations compared to the sham stimulation group (*p* = 0.03, 0.01, and <0.001, respectively). Similarly, the active PPC group exhibited a trend towards significantly higher levels of tingling, itching, and burning sensations relative to the sham stimulation group (*p* = 0.06, 0.05, and 0.07, respectively). No severe adverse events occurred.

The number of participants who guessed they were assigned to the real stimulation group was 11 (69%), 14 (82%), and 14 (74%) in the active DLPFC group, active PPC group, and sham stimulation group, respectively. The distribution across the groups demonstrated no significant variance (χ^2^_2_ = 0.90, *p* = 0.67).

## Discussion

Our study demonstrated that tDCS targeting the PPC exhibited an enhancement in working memory compared to tDCS targeting the DLPFC during concurrent cognitive task, reaching a large effect size. After the intervention, the working memory levels of the active PPC group approached those of the healthy controls. Our study also found that in the active PPC group, there was a notable augmentation in MMN amplitude, and improvements in MMN theta band measures linked to enhancements in working memory. Conversely, in both the active DLPFC group and the sham tDCS group, there were no associations between changes in working memory and changes in MMN measures or d-serine levels.

The superiority of tDCS targeting the PPC in our study suggests that the PPC may be a better neuromodulation target for enhancing working memory in schizophrenia and that it also has a causal role in working memory capacity in schizophrenia. Wang et al. [16] similarly found that in healthy individuals, tDCS targeting the PPC enhanced visual working memory capacity, but did not affect precision, and tDCS targeting the DLPFC did not change working memory capacity or precision. Their further analysis highlighted the consistency and stability of the enhancement in visual working memory capacity through tDCS applied to the PPC across individuals. Conversely, they found that the effects of tDCS targeting the DLPFC varied significantly across individuals, which our study also observed. There were no significant differences in the primary outcome between the active PPC group and the sham stimulation group in our study, which may be attributed to the low sensitivity of the spatial span test scores to the intervention. Our findings suggest that delay-estimation task capacity is more sensitive to intervention effects and might be more appropriate as a primary outcome measure. Further validation in future research is essential.

The associations between changes in working memory and changes in MMN in our study imply that enhancements in NMDAR functioning may help bolster working memory when tDCS targets the PPC during concurrent cognitive task, but seem to be non-beneficial for working memory when tDCS targets the DLPFC with concurrent cognitive task. Therefore, direct stimulation via tDCS may not necessarily improve DLPFC functioning for working memory. Moreover, a large multi-site functional magnetic resonance imaging study [63] found that as memory load increased from low to moderate, the change of DLPFC activation was higher in schizophrenic patients compared to healthy controls. This finding suggests that working memory deficits are linked with DLPFC inefficiency instead of hyper- or hypo-frontality in schizophrenia. These findings might explain results from our study and negative cognitive performance in the active DLPFC group in a large-scale tDCS trial conducted by Bulubas et al. [64]. Additionally, the active PPC group demonstrated significant cognitive effects at both week 1 and week 2 compared to the active DLPFC group, yet only at week 2 relative to the sham group. This finding might also be attributed to the complex relationship between working memory and the DLPFC dysfunction in schizophrenia.

We found that tDCS targeting the PPC with concurrent cognitive performance improved MMN amplitude in schizophrenia. Previous research has indicated that the primary benefits of high-order cognitive training in schizophrenia manifest in neurocognition and real-world functioning, with limited impact on MMN [65]. Sehatpour et al. [31] observed that augmenting training with d-serine could elevate MMN amplitude in schizophrenia, displaying a dose-dependent effect. Our finding offers initial evidence for the efficacy of enhancing cognitive training with tDCS on MMN amplitude in schizophrenia and underscores a target-dependent effect, which bears significant clinical implications.

The three groups did not differ significantly regarding improvement in other domains of neurocognition in our study. Tang et al. [66] similarly demonstrated that transcranial magnetic stimulation with individualized targets based on the parietal-hippocampal functional connection improved the visuospatial learning but did not improve other cognitive functions in individuals at clinical high risk for psychosis, as well as schizophrenic patients. tDCS increases the likelihood of neuronal activation [6], thus tDCS may preferentially regulate the brain networks activated in the task, as per the network activity-dependent model of transcranial electrical stimulation [67]. Our study demonstrated that combining tDCS with concurrent visual working memory task could improve visual working memory but not other cognitive functions, lending support to the network activity-dependent model. Likewise, Wang et al. [21] found that tDCS improved motor learning in mice only when applied during movement. This improvement of motor learning by tDCS was task-specific, and tDCS served to strengthen the neural substrates underpinning the given task during intervention. These findings suggest that combining non-specific modulation of tDCS with task-specific activation of brain networks may allow for a more precise modulation of specific brain networks, much akin to the therapeutic effect of individualized TMS based on functional connectivity.

The active DLPFC group demonstrated an advantage in ameliorating negative symptoms relative to the active PPC group in our study. However, negative symptoms were also significantly reduced in the sham group vs the active PPC group. A meta-analysis has found that cognitive training can alleviate negative symptoms [68], which might account for the observed negative symptom improvements in the group receiving sham tDCS along with concurrent cognitive tasks. Additionally, the participants in our study exhibited relatively mild negative symptoms at baseline. This factor, especially in a study with a limited sample size, could accentuate the variability in individual responses to treatment, thereby increasing the likelihood of false-positive findings. Meanwhile, the initial mildness of the negative symptoms suggests that any observed changes might hold limited clinical relevance. Nevertheless, the existence of other unidentified mechanisms influencing these results cannot be ruled out. For instance, it remains to be explored whether the PPC stimulation might disrupt the effect of cognitive training on negative symptoms. Therefore, the effects of high-definition tDCS targeting the PPC on negative symptoms warrant further investigation. Additionally, there were no severe adverse events in our study, indicating the safety and tolerability of high-definition tDCS.

## Limitations

The limitations of our study should be noted. First, despite the great challenges in recruiting clinically stable schizophrenia patients for clinical trials, our sample size was limited. Thus, our study should be considered exploratory, primarily serving as a proof-of-concept investigation. The findings need external validation with larger samples to confirm their applicability and reliability. Second, the duration of the follow-up was short. Future research with a longer follow-up period would be valuable. Furthermore, given the economic, portable, user-friendly, and safe advantages of tDCS and cognitive tasks, it is also worthwhile to investigate whether long-term use of home-based tDCS with concurrent cognitive performance can effectively sustain therapeutic effects over time. Last, it should be acknowledged that the significant practice effects might be an influential factor in the absence of notable differences between groups in the MCCB composite scores and some subdomains at week 1 and week 2. This aspect necessitates further investigation.

## Conclusions

In conclusion, our study observed that tDCS targeting the PPC as opposed to the DLPFC with simultaneous working memory task improved working memory in schizophrenia. The enhancement in working memory may be associated with an upregulation of NMDAR functioning. However, no significant differences were observed in the primary outcome between the active PPC group and the sham stimulation group. This suggests that claims of the superiority of tDCS targeting the PPC in enhancing working memory warrant further investigation and validation.

### Supplementary information

Supplementary Information

## Data Availability

The data that support the findings of this study are available on request from the corresponding author (CW).
