# Supplementary material for: Effect of transcranial direct current stimulation with concurrent cognitive performance targeting posterior parietal cortex vs prefrontal cortex on working memory in schizophrenia: a randomized clinical trial
Source: Transl Psychiatry. 2024 Jul 8;14:279. doi: 10.1038/s41398-024-02994-w (PMC11231223; doi:10.1038/s41398-024-02994-w)
Supplement: Supplementary file 1 — Supplementary Information [file 41398_2024_2994_MOESM1_ESM.doc]

**Supplementary Information**

Supplementary Methods

Content Comparison Table before and after Revision of Inclusion and Exclusion Criteria

Supplementary Figure 1. Study Flow Diagram

Supplementary Figure 2. Electrode Placement and Current Density Distributions

Supplementary Figure 3. Change in Spatial Span Test Scores

Supplementary Figure 4. Average MMN Waveforms at Three Time Points across the Three Groups

Supplementary Figure 5. Correlation between the Change in MMN Theta Intertrial Coherence at Week 1 and the Changes in the Spatial Span Test Scores at Week 2

Supplementary Table 1. Comparison of Baseline Cognitive Domains Between Patients and Healthy Controls

Supplementary Table 2. Effects on PANSS and CDSS Scores

Supplementary Table 3. Effects on MMN and D-serine

Supplementary Table 4. Side Effects of tDCS

Supplementary Table 5. Spearman Correlations between Changes in MMN, D-serine and Changes in the Spatial Span Test Scores

Supplementary References

**Supplementary Methods**

**Exclusion Criteria**

The exclusion criteria consisted of the followings: cochlear implantation or other metal implants anywhere in the body, color blindness or color weakness, pregnancy or lactation in women, a history of severe physical illness, unstable mental state, a history of alcohol or drug abuse or dependency during the last 6 months, daily sedative hypnotic drug doses exceeding 1mg of lorazepam or equivalent, daily anticholinergic medication exceeding 10mg of benztropine or equivalent; recent treatment with stimulants, electroconvulsive therapy, electromagnetic stimulation treatment or cognitive training within the past 6 months, repeated exposures to the MATRICS Consensus Cognitive Battery (MCCB) and/or other cognitive test batteries within the past 12 weeks, inability or unwillingness to cooperate.

**Short Form of the Chinese Version of the Wechsler Adult Intelligence Scale**

The intelligence quotient (IQ) was assessed with a short form of the Chinese version of the Wechsler Adult Intelligence Scale (WAIS-RC), which included tests on information, similarities, picture completion, and block design [1]. Although there was a risk of overestimation [2], the short form of the WAIS-RC could be suitable for a rough estimate of IQ [3].

**MCCB and Digit Sequencing Task**

As the benchmark for cognitive assessment in schizophrenia-related clinical trials, the MCCB is endorsed by the U.S. Food and Drug Administration [4]. The Chinese version of the MCCB [5] encompasses seven cognitive domains and nine subtests: (1) speed of processing, using trail making test: part A, symbol coding, and animal naming; (2) attention/vigilance, using continuous performance test-identical pairs; (3) working memory, using spatial span test; (4) verbal learning, using the Hopkins verbal learning test-revised; (5) visual learning, using the brief visuospatial memory test-revised; (6) reasoning and problem solving, using the mazes subtest; (7) social cognition, using the Mayer-Salovey-Caruso emotional intelligence test: managing emotions. For this study, the first six cognitive domains were utilized to calculate the overall neurocognitive score. To compensate for the Chinese MCCB’s lack of assessment for verbal working memory [5], we added the digit sequencing task from the Brief Assessment of Cognition in Schizophrenia (BACS) [6].

**Color Delay-estimation Task**

The color delay-estimation task [7] involves the presentation of either one or three colored squares at a time. Results were calculated based on trials in which three squares were presented in this study. The distribution of response errors was analyzed using the standard mixture model [8] in the MemToolbox [9]. The reciprocal of the standard deviation represented working memory precision, while parameter g reflected the guess probability within response errors. The product of memory load and (1-g) is indicative of working memory capacity.

**Stroop with Adaptive Response Deadline**

In the Stroop with adaptive response deadline [10, 11], the response deadline for each trial is dynamically adjusted based on the accuracy and duration of the response in the preceding incongruent trial. The test comprises four blocks of 72 trials each, totaling 288 trials. The final derived metric is the average response time across the last four reversals. A reversal was defined as a trial where the change in the deadline contrasts with the change in the previous trial.

**Mismatch Negativity Data Collection**

Mismatch negativity (MMN) was elicited using the classical Oddball paradigm, which was composed of 90% standard stimuli and 10% duration-deviant stimuli. The standard stimuli were pure tones set at 1000 Hz, 50 ms, and 75 dB, while the duration-deviant stimuli were pure tones set at 1000 Hz, 100 ms, and 75 dB. The task consisted of three blocks, with each comprising 225 standard stimuli and 25 deviant stimuli, cumulatively yielding 750 trials. Data was collected using the 128-channel high-density EEG system from EGI (Electrical Geodesics, Inc., USA). The sampling rate was set at 1000 Hz, with E129 serving as the online reference electrode. Saline-based electrode caps were used, and the impedance of each electrode remained below 50 kΩ. Participants were instructed to keep relaxed and still in a soundproof and electromagnetically shielded chamber.

**Mismatch Negativity Data Processing**

MMN data were processed using the EEGLAB 2022.0 toolkit (http://sccn.ucsd.edu/eeglab/) and custom-written scripts based on MATLAB R2020b (The MathWorks Inc., Natick, MA, USA). Analysts were blind to group assignments of participants.

Pre-processing steps: (1) Data underwent 0.5 Hz high-pass filtering, 30 Hz low-pass filtering, and a notch filter between 48-52 Hz to eliminate line noise (Basic FIR filter); (2) The sampling rate was reduced to 500 Hz; (3) Continuous data were segmented based on stimulus onset, extracting epochs from -100 ms to 500 ms. Subsequent baseline correction was performed by subtracting the mean amplitude of the pre-stimulus interval lasting 100 ms; (4) Drifting or problematic electrodes were replaced, and segments exhibiting significant drift, muscular artifacts, or line noise were excluded; (5) A global average re-referencing strategy was applied; (6) Independent component analysis was utilized to identify and remove artifact components including blinks, lateral eye movements, muscular activities, cardiac signals, and malfunctioning electrodes; (7) Any segments with amplitudes exceeding ±100μV were omitted.

Time domain analysis: Electrode E6, corresponding to the 10-20 system's FCz, was selected for analysis. MMN waveform was derived by subtracting waveform elicited by standard stimuli from that of the deviant stimuli. MMN amplitude was determined by extracting the peak negative amplitude between 140-240 ms from MMN waveform. All these steps were performed using custom-written scripts.

Time-frequency analysis: Data were downsampled to 250 Hz to reduce file size and computational time. Theta band (4-7 Hz) was selected, and Morlet wavelet transformation was applied to compute power and intertrial coherence for each participant under deviant stimuli. MMN theta power and theta intertrial coherence were defined as the peak mean power and intertrial coherence within the theta band during the 140-240 ms interval following the deviant stimuli. These steps utilized scripts authored by Morales S et al [12].

**D-serine Assay**

Serum samples were collected at baseline, week 1, and week 2. Samples from the same participant were drawn at consistent times of the day (± 2 h). Post-centrifugation, serum samples were stored at -80℃. Serum concentrations of D-serine were obtained using the enzyme-linked immunosorbent assay technique, facilitated by the Varioskan Flash (ThermoFisher Scientific, MA, USA). The personnel performing the assays remained blind to group assignments of participants.

**N-back Test During Each tDCS Session**

During each tDCS session, participants undertook an adaptive n-back test [13], where blue squares appeared at random locations on the screen. Participants were required to press the ‘A’ key when a square reappeared at the same location as one shown in the preceding trial, specifically in 1-back, 2-back, 3-back, or more challenging sequences. The difficulty level dynamically adapted to the participants' response accuracy: it increased by one level for accuracy at or above 90%, decreased by one level for accuracy at or below 70%, and remained the same otherwise.


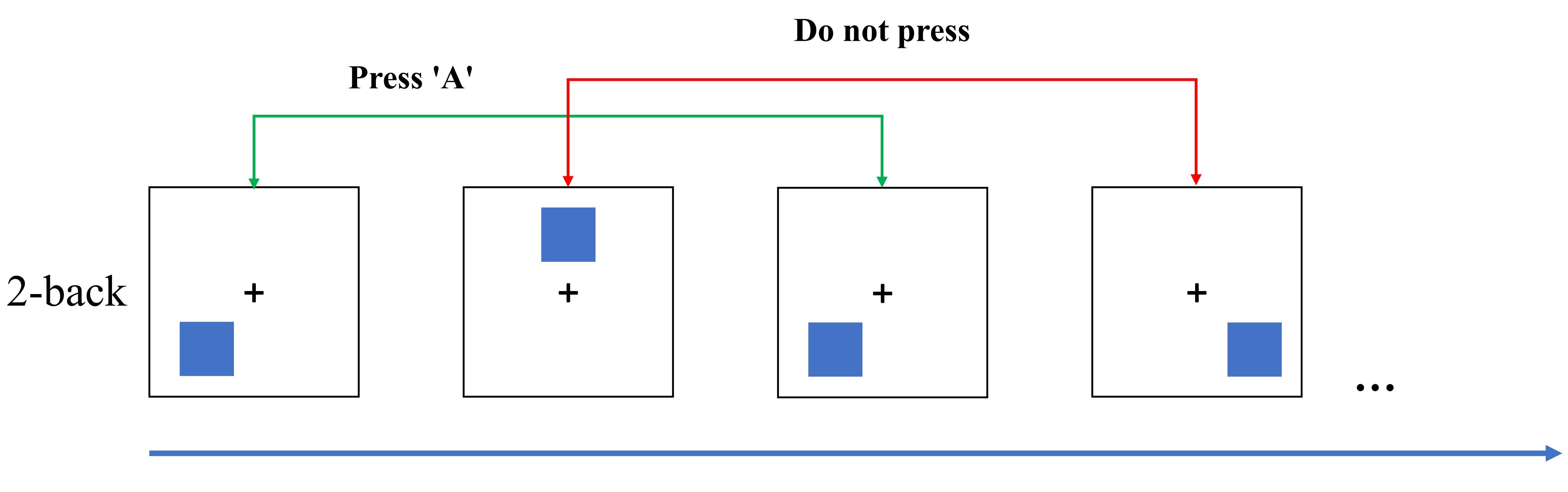


**Content Comparison Table before and after Revision of Inclusion and Exclusion Criteria**

| Revised part | Original description in September 2021 | New description in November 2021 |
| --- | --- | --- |
| Inclusion criteria | 2. Patients aged 18-45 years, Han Chinese, with right handedness, years of education ≥ 9 ((junior high school or above); | 2. Patients aged 18-50 years, with right handedness, years of education ≥ 8; |
|  | 6. Patients who got T score 35-45 in the spatial span test of MCCB; | 6. Patients who got T score 30-50 in the spatial span test of MCCB; |
|  | 7. Patients must take the same type of antipsychotic medications during the study period. | 7. Patients who are clinically stable, maintained on current antipsychotic and concomitant medications for at least 6 weeks, with no change in antipsychotic dose for at least 2 weeks. Patients must take the same type and dose of antipsychotic medications during the study period. |
| Exclusion criteria | 7. Patients taking first-generation antipsychotic drugs, cognitive enhancers (such as methylphenidate), anti-dementia drugs; | 7. Patients taking sedative-hypnotic drugs at a dose greater than lorazepam 1 mg / day or equivalent; |
|  | 8. Patients taking benzodiazepines at a dose greater than 10mg of diazepam / 2mg of lorazepam per day or equivalent; | 8. Patients taking anticholinergic drugs at a dose greater than trihexyphenidyl 10 mg / day or equivalent; |
|  | 9. Patients taking benzodiazepines or other hypnotics within 6 hours before the cognitive assessment; | 9. Patients taking stimulants (e.g., methylphenidate, dextroamphetamine, modafinil), or receiving electroconvulsive therapy or electromagnetic stimulation or cognitive training within 6 months; |
|  | 10. Patients receiving electroconvulsive therapy or electromagnetic stimulation therapy within 6 months; | 10. Patients receiving repeat assessments using MCCB and/or other cognitive test kits within 12 weeks (i.e., single assessment is not excluded); |

Abbreviations: MCCB, MATRICS Consensus Cognitive Battery.

**
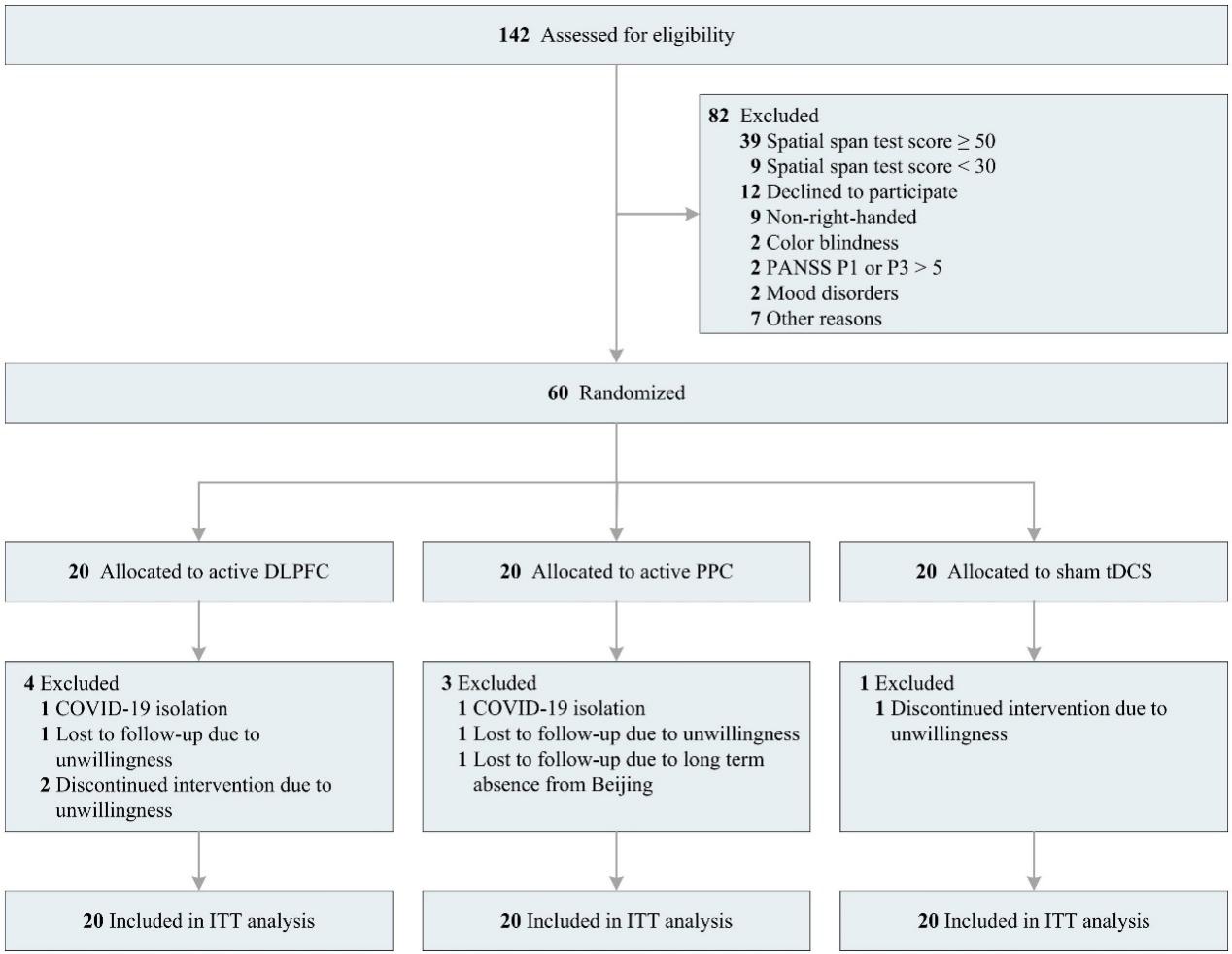
**

**Supplementary Figure 1.** Study flow diagram.

Abbreviations: PANSS, Positive and Negative Syndrome Scale; DLPFC, dorsolateral prefrontal cortex; PPC, posterior parietal cortex; tDCS, transcranial direct current stimulation; ITT, intention to treat.


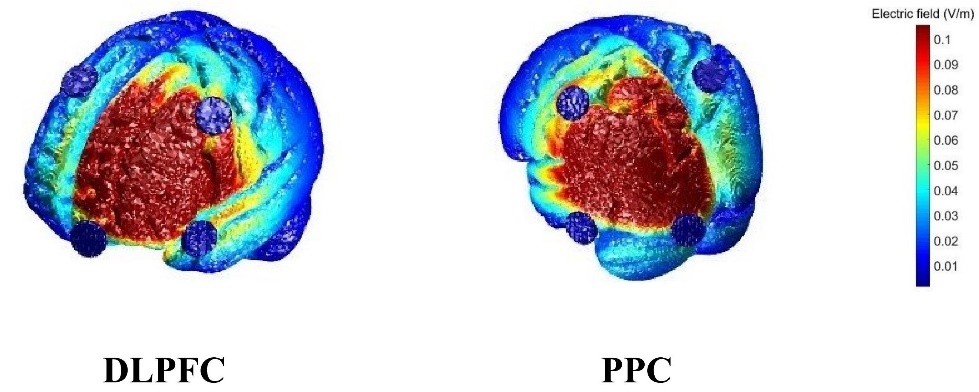


**Supplementary Figure 2.** Electrode placement and current density distributions.

Abbreviations: DLPFC, dorsolateral prefrontal cortex; PPC, posterior parietal cortex.

**
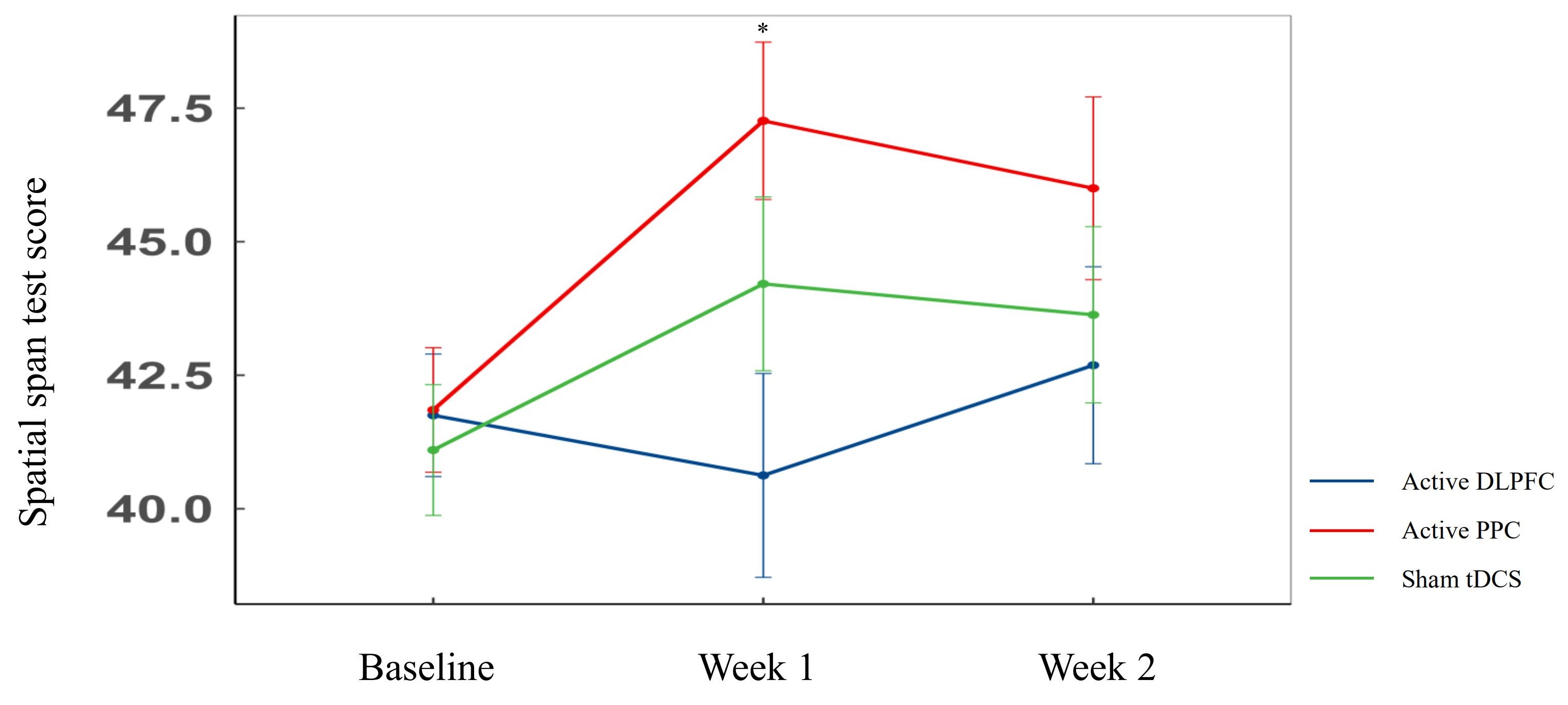
****Supplementary Figure 3.** Change in Spatial Span Test Scores.

Abbreviations: DLPFC, dorsolateral prefrontal cortex; PPC, posterior parietal cortex.

**
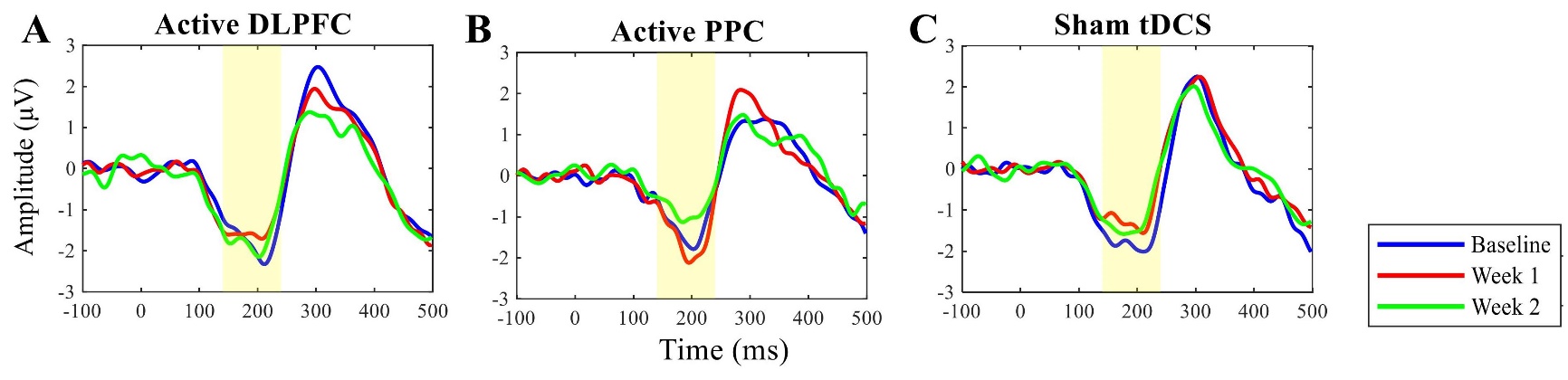
**

**Supplementary Figure 4.** Average MMN waveforms at three time points across the three groups.

Abbreviations: MMN, mismatch negativity; DLPFC, dorsolateral prefrontal cortex; PPC, posterior parietal cortex; tDCS, transcranial direct current stimulation.

**
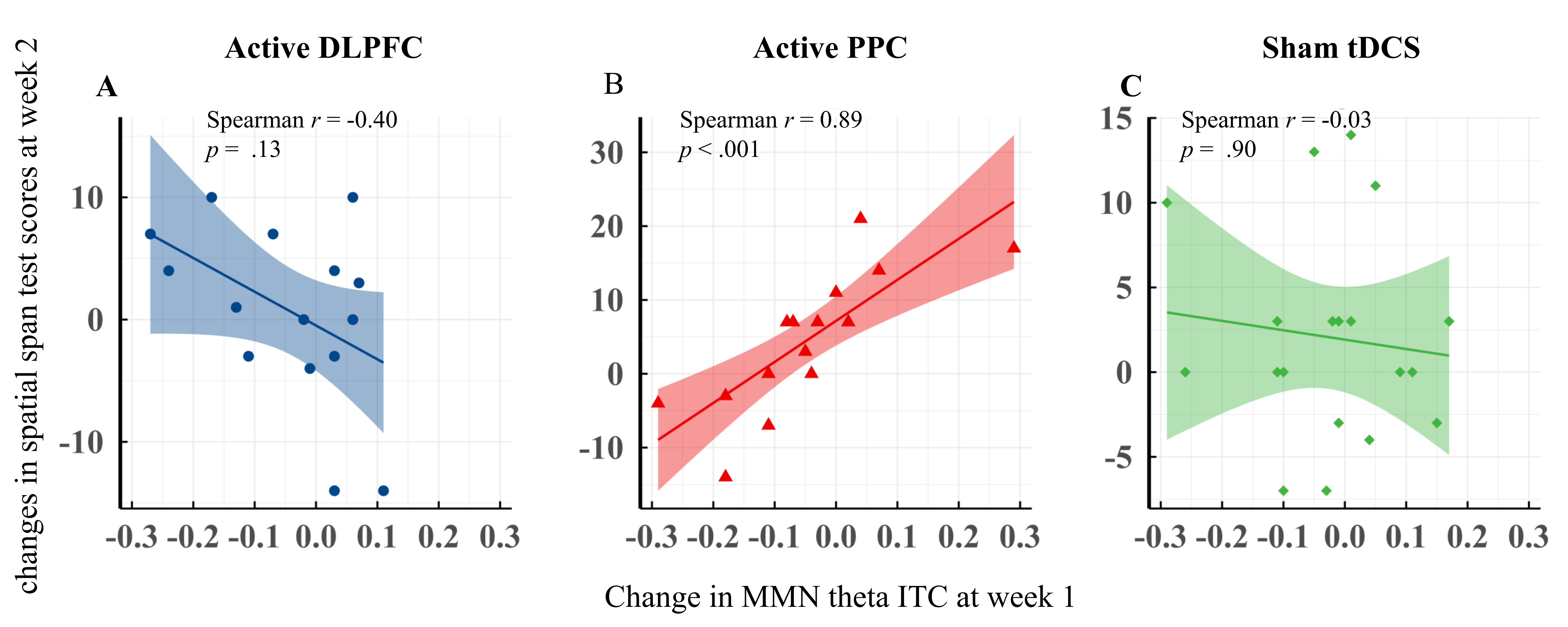
**

**Supplementary Figure 5.** Correlation between the change in MMN theta intertrial coherence at week 1 and the changes in the spatial span test scores at week 2. This figure illustrates the correlations between the change in MMN theta intertrial coherence at week 1 from baseline and the changes in the spatial span test scores at week 2 from baseline in the active DLPFC group **(A),** the active PPC group **(B),** and the sham tDCS group **(C)**.

Abbreviations: MMN, mismatch negativity; ITC, intertrial coherence; DLPFC, dorsolateral prefrontal cortex; PPC, posterior parietal cortex; tDCS, transcranial direct current stimulation.

Supplementary Table 1. Comparison of Baseline Cognitive Domains Between Patients and Healthy Controls.

|  | Patients (n = 60) | Controls (n = 35) |  |  |  |
| --- | --- | --- | --- | --- | --- |
| Characteristic | Mean (SD) | Mean (SD) | Statistic | *df* | *p* |
| Women, No. (%) | 34 (57) | 16 (46) | *χ²* = 1.06 | 1 | 0.30 |
| Age, y | 33.22 (7.44) | 31.11 (5.73) | *t* = 1.54 | 86 | 0.13 |
| Education, y | 14.64 (3.55) | 15.8 (3.48) | *t* = 1.55 | 93 | 0.13 |
| IQ | 104.09 (10.51) | 115.96 (10.41) | *t* = 5.33 | 93 | < 0.001 |
| Spatial span test | 41.57 (5.20) | 51.14 (10.23) | *t* = 5.17 | 44 | < 0.001 |
| MCCB speed of processing | 38.27 (10.85) | 54.42 (9.08) | *t* = 7.42 | 93 | < 0.001 |
| MCCB attention | 46.92 (10.41) | 52.11 (9.91) | *t* = 2.39 | 93 | 0.02 |
| MCCB verbal learning | 38.02 (10.41) | 52.49 (11.64) | *t* = 6.26 | 93 | < 0.001 |
| MCCB visual learning | 41.92 (9.96) | 54.37 (7.47) | *t* = 6.91 | 87 | < 0.001 |
| MCCB reasoning and problem solving | 44.2 (10.99) | 50.26 (11.58) | *t* = 2.54 | 93 | 0.01 |

Abbreviations: IQ, intelligence quotient; MCCB, MATRICS Consensus Cognitive Battery.

**Supplementary Table 2. Effects on PANSS and CDSS Scores.**

| Outcome | tDCS Group, Mean (SD) | | | *p* | Active DLPFC  vs Active PPC | | Active DLPFC  vs Sham tDCS | | Active PPC  vs Sham tDCS | |
| --- | --- | --- | --- | --- | --- | --- | --- | --- | --- | --- |
|  | Active DLPFC  (n = 20) | Active PPC  (n = 20) | Sham tDCS  (n = 20) |  |  |  |  |  |  |  |
|  |  |  |  |  | *p* | Cohen’s *d* (95% CI) | *p* | Cohen’s *d* (95% CI) | *p* | Cohen’s *d* (95% CI) |
| Change at week 1 *^a^* |  |  |  |  |  |  |  |  |  |  |
| PANSS Positive | 0.19 (1.17) | -0.53 (2.52) | 0.32 (1.89) | .43 | > .99 | -0.36 (-1.03 - 0.31) | .66 | 0.08 (-0.58 - 0.75) | .25 | 0.38 (-0.26 - 1.02) |
| PANSS Negative | -0.75 (1.77) | 0.47 (2.87) | -0.89 (2.05) | .15 | .15 | 0.50 (-0.17 - 1.18) | .80 | -0.07 (-0.74 - 0.59) | .09 | -0.55 (-1.19 - 0.10) |
| PANSS General | -1.69 (3.22) | -0.58 (1.74) | -0.16 (2.69) | .15 | .13 | 0.44 (-0.23 - 1.11) | .09 | 0.52 (-0.16 - 1.20) | .58 | 0.19 (-0.45 - 0.82) |
| PANSS total | -2.25 (3.59) | -0.63 (5.28) | -0.74 (4.60) | .48 | .24 | 0.35 (-0.32 - 1.02) | .27 | 0.36 (-0.31 - 1.03) | .96 | -0.02 (-0.66 - 0.61) |
| Change at week 2 *^b^* |  |  |  |  |  |  |  |  |  |  |
| PANSS Positive | 0.13 (1.67) | 0.35 (2.00) | -0.58 (1.07) | .26 | .74 | 0.12 (-0.56 - 0.80) | .47 | -0.52 (-1.19 - 0.16) | .10 | -0.59 (-1.26 - 0.08) |
| PANSS Negative | -1.38 (2.36) | 0.24 (1.64) | -2.00 (2.94) | .02 | .04 | 0.80 (0.09 - 1.51) | .32 | -0.23 (-0.90 - 0.44) | .009 | -0.93 (-1.62 - -0.24) |
| PANSS General | -2.56 (3.69) | 0.00 (3.82) | -0.47 (3.78) | .07 | .06 | 0.68 (-0.02 - 1.38) | .06 | 0.56 (-0.12 - 1.24) | .73 | -0.12 (-0.78 - 0.53) |
| PANSS total | -3.81 (4.71) | 0.59 (4.96) | -3.05 (5.70) | .03 | .01 | 0.91 (0.19 - 1.63) | .52 | 0.14 (-0.52 - 0.81) | .054 | -0.68 (-1.35 - 0.00) |
| CDSS (In-transformed) | -0.04 (0.17) | -0.06 (0.22) | -0.02 (0.16) | .54 | .70 | -0.10 (-0.78 to 0.58) | .49 | 0.12 (-0.54 to 0.79) | .29 | 0.21 (-0.45 to 0.87) |

Abbreviations: PANSS, Positive and Negative Syndrome Scale; CDSS, Calgary Depression Scale for Schizophrenia; DLPFC, dorsolateral prefrontal cortex; PPC, posterior parietal cortex; tDCS, transcranial direct current stimulation. *^a^* for PANSS at week 1, the sample size was 16 (active DLPFC group), 19 (active PPC group) and 19 (sham tDCS group). *^b^* for PANSS and CDSS at week 2, the sample size was 16 (active DLPFC group), 17 (active PPC group) and 19 (sham tDCS group).

**Supplementary Table 3. Effects on MMN and D-serine.**

| Measures | tDCS Group, Mean (SD) | | | *p* | Active DLPFC  vs Active PPC | | Active DLPFC  vs Sham tDCS | | Active PPC  vs Sham tDCS | |  |
| --- | --- | --- | --- | --- | --- | --- | --- | --- | --- | --- | --- |
|  | Active DLPFC  (n = 20) | Active PPC  (n = 20) | Sham tDCS  (n = 20) |  |  |  |  |  |  |  |  |
|  |  |  |  |  | *p* | Cohen’s *d*  (95% CI) | *p* | Cohen’s *d*  (95% CI) | *p* | Cohen’s *d*  (95% CI) | |
| Change in score at week 1 |  |  |  |  |  |  |  |  |  |  | |
| MMN amplitude (In-transformed) *^a^* | 0.09 (0.19) | -0.10 (0.30) | 0.20 (0.34) | .007 | .04 | -0.75  (-1.44 to -0.05) | .16 | 0.39 (-0.28 to 1.06) | .006 | 0.93 (0.26 to 1.61) | |
| MMN theta power (In-transformed) *^a^* | -0.003 (0.10) | -0.001 (0.08) | -0.01 (0.08) | .92 | .75 | 0.02 (-0.65 to 0.70) | .95 | -0.08 (-0.74 to 0.59) | .68 | -0.11 (-0.76 to 0.53) | |
| MMN theta intertrial coherence (In-transformed) *^a^* | -0.003 (0.01) | -0.005 (0.01) | -0.002 (0.01) | .69 | .51 | -0.20 (-0.88 to 0.48) | .91 | 0.10 (-0.57 to 0.77) | .44 | 0.30 (-0.35 to 0.95) | |
| D-serine (In-transformed) *^b^* | 0.09 (0.25) | 0.07 (0.22) | -0.03 (0.18) | .23 | .83 | -0.09 (-0.78 to 0.61) | .14 | -0.56 (-1.26 to 0.14) | .13 | -0.50 (-1.17 to 0.17) | |
| Change in score at week 2 |  |  |  |  |  |  |  |  |  |  | |
| MMN amplitude (In-transformed) *^c^* | 0.06 (0.23) | 0.06 (0.17) | 0.18 (0.35) | .25 | .44 | 0 (-0.70 to 0.70) | .15 | 0.40 (-0.27 to 1.07) | .30 | 0.42 (-0.26 to 1.11) | |
| MMN theta power (In-transformed) *^c^* | -0.04 (0.08) | -0.02 (0.05) | -0.04 (0.07) | .60 | .35 | 0.30 (-0.41 to 1.01) | .77 | 0 (-0.67 to 0.67) | .38 | -0.32 (-1.00 to 0.36) | |
| MMN theta intertrial coherence (In-transformed) *^c^* | -0.005 (0.01) | -0.001 (0.01) | -0.002 (0.01) | .80 | .64 | 0.40 (-0.31 to 1.11) | .52 | 0.30 (-0.37 to 0.97) | .71 | -0.10 (-0.78 to 0.58) | |
| D-serine (In-transformed) *^d^* | 0.07 (0.16) | 0.05 (0.27) | 0.01 (0.16) | .70 | .92 | -0.09 (-0.81 to 0.63) | .33 | -0.38 (-1.07 to 0.32) | .55 | -0.19 (-0.87 to 0.50) | |

Abbreviations: tDCS, transcranial direct current stimulation; MMN, mismatch negativity; DLPFC, dorsolateral prefrontal cortex; PPC, posterior parietal cortex. *^a^* for MMN at week 1, the sample size was 16 (active DLPFC group),18 (active PPC group) and 19 (sham tDCS group). *^b^* for D-serine at week 1, the sample size was 15 (active DLPFC group), 17 (active PPC group) and 18 (sham tDCS group). *^c^* for MMN at week 2, the sample size was 16 (active DLPFC group), 15 (active PPC group) and 19 (sham tDCS group). *^d^* for D-serine at week 2, the sample size was 15 (active DLPFC group), 15 (active PPC group) and 18 (sham tDCS group).

**Supplementary Table 4. Side Effects of tDCS.**

| Adverse effects questionnaire | tDCS Group, Mean (SD) | | |  | Active DLPFC  vs Active PPC | Active DLPFC  vs Sham tDCS | Active PPC  vs Sham tDCS |
| --- | --- | --- | --- | --- | --- | --- | --- |
|  | Active DLPFC  (n = 17) | Active PPC  (n = 20) | Sham tDCS  (n = 19) | *p* |  |  |  |
|  |  |  |  |  | *p* | *p* | *p* |
| Headache | 1.11 (0.28) | 1.03 (0.06) | 1.12 (0.21) | .34 | .23 | .95 | .19 |
| Neck pain | 1.02 (0.05) | 1.01 (0.02) | 1.03 (0.10) | .47 | .57 | .53 | .22 |
| Scalp pain | 1.49 (0.54) | 1.28 (0.35) | 1.19 (0.29) | .08 | .11 | .03 | .54 |
| Tingling | 1.86 (0.54) | 1.72 (0.61) | 1.41 (0.30) | .03 | .41 | .01 | .06 |
| Itching | 1.06 (0.13) | 1.09 (0.17) | 1.01 (0.02) | .14 | .53 | .21 | .05 |
| Burning sensation | 1.63 (0.58) | 1.35 (0.43) | 1.10 (0.18) | .002 | .05 | < .001 | .07 |
| Skin redness | 1.34 (0.39) | 1.44 (0.45) | 1.54 (0.43) | .38 | .51 | .16 | .44 |
| Sleepiness | 1.39 (0.49) | 1.50 (0.38) | 1.39 (0.42) | .67 | .44 | .96 | .45 |
| Trouble concentrating | 1.28 (0.37) | 1.34 (0.36) | 1.42 (0.41) | .58 | .65 | .30 | .54 |
| Acute mood change | 1.02 (0.04) | 1.02 (0.05) | 1.01 (0.02) | .38 | .80 | .20 | .28 |

Abbreviations: tDCS, transcranial direct current stimulation; DLPFC, dorsolateral prefrontal cortex; PPC, posterior parietal cortex.

**Supplementary Table 5.** Spearman Correlations between Changes in MMN, D-serine and Changes in the Spatial Span Test Scores.

| Group | Change in measures from baseline | MMN amplitude at week 1 | MMN theta power at week 1 | MMN theta ITC at week 1 | D-serine at week 1 |
| --- | --- | --- | --- | --- | --- |
| Active DLPFC | Sample size | 16 | 16 | 16 | 15 |
|  | Spatial span test score at week 1 | 0.21 | -0.51 | -0.19 | -0.08 |
|  | Spatial span test score at week 2 | 0.17 | -0.42 | -0.40 | -0.12 |
| Active PPC | Sample size | 16-18 | 16-18 | 16-18 | 15-17 |
|  | Spatial span test score at week 1 | -0.16 | 0.15 | 0.38 | 0.08 |
|  | Spatial span test score at week 2 | -0.20 | 0.07 | 0.89^***^ | 0.07 |
| Sham tDCS | Sample size | 19 | 19 | 19 | 18 |
|  | Spatial span test score at week 1 | 0.10 | -0.19 | -0.05 | -0.32 |
|  | Spatial span test score at week 2 | -0.11 | 0.39 | -0.03 | -0.47 |

Abbreviations: MMN, mismatch negativity; ITC, intertrial coherence; DLPFC, dorsolateral prefrontal cortex; PPC, posterior parietal cortex; tDCS, transcranial direct current stimulation. *** *p* < .001 after FDR correction.

**Supplementary References**

1. Pang YX, Zhang J, Yang CL, Cang Y, Wang XL. Application of WAIS-RC short forms and adult intelligence disability scale in mental impairment assessment. Fa Yi Xue Za Zhi. 2011;27**:**189-92. (in Chinese)

2. Jiang L. Analysis of the changes of short forms of WAIS-RC within 20 years. Chinese Journal of Clinical Psychology. 2006;14**:**117, 121-22. (in Chinese)

3. Bo Q, Mao Z, Tian Q, Yang N, Li X, Dong F, et al. Impaired sensorimotor gating using the acoustic prepulse inhibition paradigm in individuals at a clinical high risk for psychosis. Schizophr Bull. 2021;47**:**128-37.

4. Buchanan RW, Keefe RS, Umbricht D, Green MF, Laughren T, Marder SR. The FDA-NIMH-MATRICS guidelines for clinical trial design of cognitive-enhancing drugs: what do we know 5 years later? Schizophr Bull. 2011;37**:**1209-17.

5. Shi C, Kang L, Yao S, Ma Y, Li T, Liang Y, et al. The MATRICS consensus cognitive battery (MCCB): Co-norming and standardization in China. Schizophr Res. 2015;169**:**109-15.

6. Keefe RS, Goldberg TE, Harvey PD, Gold JM, Poe MP, Coughenour L. The brief assessment of cognition in schizophrenia: reliability, sensitivity, and comparison with a standard neurocognitive battery. Schizophr Res. 2004;68**:**283-97.

7. Zhao YJ, Ma T, Zhang L, Ran X, Zhang RY, Ku Y. Atypically larger variability of resource allocation accounts for visual working memory deficits in schizophrenia. Plos Comput Biol. 2021;17**:**e1009544.

8. Zhang W, Luck SJ. Discrete fixed-resolution representations in visual working memory. Nature. 2008;453**:**233-35.

9. Suchow JW, Brady TF, Fougnie D, Alvarez GA. Modeling visual working memory with the MemToolbox. J Vis. 2013;13:9.

10. Draheim C, Tshukara JS, Engle RW. Replication and extension of the toolbox approach to measuring attention control. Behav Res Methods. 2023**:**1-23.

11. Draheim C, Tsukahara JS, Martin JD, Mashburn CA, Engle RW. A toolbox approach to improving the measurement of attention control. J Exp Psychol Gen. 2021;150**:**242-75.

12. Morales S, Bowers ME. Time-frequency analysis methods and their application in developmental EEG data. Dev Cogn Neurosci. 2022;54**:**101067.

13. Working Memory and Plasticity Lab. https://wmp.education.uci.edu/software/. Accessed 10 July 2023.
